# Joint modeling of longitudinal and time‐to‐event data for dynamic disease risk prediction using proteomics

**DOI:** 10.1002/pro.70621

**Published:** 2026-05-18

**Authors:** Markus Lindén, Tea Ammunét, Tommi Välikangas, Laura L. Elo, Tomi Suomi

**Affiliations:** ^1^ Turku Bioscience Centre University of Turku and Åbo Akademi University Turku Finland; ^2^ Institute of Biomedicine, University of Turku Turku Finland

**Keywords:** joint model, longitudinal, risk prediction, survival analysis, type 1 diabetes

## Abstract

Biomedical studies increasingly incorporate longitudinal data, enabling us to track individual disease processes over time at the molecular level, and to discover associations of the molecular profiles with the outcome of interest, such as the onset of a disease. Despite the potential of statistical methods that jointly model longitudinal and time‐to‐event data, they have not yet been widely adopted in high‐throughput omics studies. Therefore, we evaluated multiple approaches for joint modeling of longitudinal and time‐to‐event data, and we introduce a joint modeling strategy for longitudinal proteomics studies. The focus is on assessing the utility of the methods in predicting the dynamic disease risk of an individual from longitudinal proteome profiles. To benchmark the methods, we used a range of simulated datasets that reflected real proteome profiles with varying complexities. Our results clearly demonstrated the advantages of the longitudinal methods over conventional Cox proportional hazards models with single time point studies. This was further supported by re‐analysis of data from a proteomics study of early type 1 diabetes prediction, where we discovered new early candidate proteins associated with the disease onset that were not detected in the original study.

## 1 INTRODUCTION

Personalized medicine has gained popularity as a means to enable more individualized clinical decisions. This often involves risk predictions based on an individual's biological profile, which can be monitored over time to assess, for instance, the risk of disease onset or adverse events related to treatment. Traditionally, risk estimations have been repeated several times following updates to the individual's biological measurements. However, instead of using traditional time‐to‐event (survival) models based on data from a single time point, it has been suggested that a longitudinal approach that considers the full history of these measurements would be more effective (Baart et al., 2021; Yuen & Mackinnon, 2016).

Predictive modeling frameworks exist for longitudinal data, implementing both machine learning classifiers and joint models of longitudinal and time‐to‐event data, as reviewed for example by (Bull et al., 2020). In particular, joint models have gained attention during the last decade. Joint models combine models of longitudinal trends with models of time‐to‐event outcomes. Different model structures, associations between the two types of models, and methods to estimate the model parameters have been implemented and extensively reviewed, for example, by (Hickey et al., 2016, 2018a), (Alsefri et al., 2020; Papageorgiou et al., 2019). Recent advancements have been made in joint latent class models (Sun et al., 2019), landmarking models, and partly conditional models (Devaux et al., 2022; Ferrer et al., 2019), as well as related random forest‐based solutions (Zhao et al., 2020) and two‐stage approaches (Mauff et al., 2020). The advances have been particularly useful in implementing dynamic prediction models and evaluating the performance of joint models in clinical data (Papageorgiou et al., 2019). Although the main focus of this study is the joint modeling (JM) of time‐to‐event and longitudinal data, there are other forms, such as the simultaneous modeling of multiple longitudinal outcomes (Ouko et al., 2025).

Current applications of joint models in medical research are dominated by studies involving a single or few known features from medical assays (Alsefri et al., 2020). However, disease progression and status are often functions of many factors, and early disease indicators often remain unknown. High‐throughput molecular omics technologies offer excellent opportunities to profile a multitude of molecular features at once, enabling the discovery of novel early disease indicators and opportunities for preventive measures before disease onset. However, the challenge with such omics data is to capture as much data as possible without suffering too much from the curse of dimensionality. In general, joint models are computationally complex, and including multivariate models even for a handful of features has called for improvements in the methods (Mauff et al., 2020; Zhao et al., 2020). Current multivariate joint models require the number of features to be much smaller than that of subjects (Zhao et al., 2020), calling for feature selection or dimensionality reduction prior to analysis. There has been an attempt to reduce the computational complexity of joint models in high‐dimensional settings by dividing the longitudinal biomarkers into pairs, calculating the joint models for these pairs that are each modeled jointly with the time‐to‐event outcome, and then pooling the results together (De Witte et al., 2025). Some recent works, such as jmBIG (Bhattacharjee et al., 2024), aim to assist in the application of joint models to large healthcare datasets through automation and parallelization. In addition, especially proteomics data often contains a considerable proportion of missing values, and regarding joint models, there have been efforts to assess the impact of different imputation methods for missing values in the covariates and responses (Bhattacharjee et al., 2020).

Due to challenges of joint models with high‐dimensional longitudinal omics data, omics applications have not been widely explored under the JM framework, with only a few exceptions. For instance, (Liu et al., 2019) introduced a Bayesian JM framework where data were first reduced to a low number of latent variables through a factor analysis model. The framework was then used to predict the survival of patients with idiopathic pulmonary fibrosis using three latent variables obtained from the expression profiles of 55 genes linked to survival outcomes. In another study, (Thomas et al., 2020) selected seven proteins based on differential abundance at three separate time points and modeled them under the multivariate JM framework with JMbayes (Rizopoulos, 2016) to predict progression‐free survival of patients receiving neoadjuvant therapy for locally advanced rectal cancer. Additionally, (Canouil et al., 2018) employed the JM framework (Rizopoulos, 2010) as well as a computationally faster approximation method to investigate the association of single nucleotide polymorphisms with the risk of developing type 2 diabetes or increased fasting plasma glucose level. Finally, (Gisby et al., 2021) used the JM framework (Rizopoulos, 2010) to identify 69 circulating proteins that were associated with the risk of COVID‐19‐related death among patients with end‐stage kidney disease.

**FIGURE 1:**
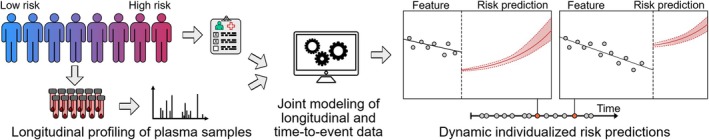
Schematic illustration of the joint modeling framework with longitudinal profiling of human plasma proteins to predict dynamic individual disease risks. First, a joint model is fitted separately for each protein using automated model selection. Proteins that show a significant association with the disease risk are then further prioritized based on their ability to predict disease outcomes. This evaluation is carried out by assessing the ability of each protein to predict the disease status of an individual by utilizing varying longitudinal follow‐up times prior to disease onset, quantified by the area under the ROC curve. Finally, the selected proteins are visualized over time.

Although there have been a few trailblazers, the overall suitability of current JM approaches on longitudinal proteomics data remains far from resolved. In addition to the high dimensionality of the data, the complexity of the data has been shown to affect the performance of joint models (Rappl et al., 2021). Therefore, it is important to pay attention to the application of joint models to omics data. The aim of this study was to explore the utility of the JM framework on research questions involving proteomics. In particular, we aimed to answer the following questions: (1) How do longitudinal joint models compare to Cox proportional hazard (CoxPH) models in predicting events? (2) What are the benefits and costs related to using joint models? (3) Can we identify promising new disease indicators from proteomics data using longitudinal joint models? To achieve these goals, we investigated the effects of different data scenarios and the number of longitudinal data points on model performance. We used both simulated data, which covered scenarios from low signal detectability to highly diverging expression patterns, and real proteomics data from a study on the early development of type 1 diabetes (Liu et al., 2018). Additionally, we present a practical JM strategy for longitudinal proteomics studies, with emphasis on predicting the dynamic disease risk of an individual from the longitudinal proteome profiles (Figure 1).

## 2 RESULTS

### 2.1 Modeling approach impacts accuracy of longitudinal prediction of time‐to‐event outcomes across simulated scenarios

To evaluate the effectiveness of different modeling approaches for predicting time‐to‐event outcomes from longitudinal data, we examined four approaches: maximum likelihood based JM (Rizopoulos, 2010), JM based on latent Gaussian processes (joineRML) (Hickey et al., 2018a), joint latent class mixed models (lcmm) (Proust‐Lima et al., 2016), and partly conditional Cox models (PCCox). Except for PCCox, these models are based on a generalized linear mixed model combined with a survival model using an association structure. PCCox, on the other hand, extends the conventional CoxPH model by conditioning the hazard on the covariate estimates through time. As a baseline reference model, we used the standard CoxPH model, which only used data from the last available time point.

To systematically assess the modeling approaches in diverse types of scenarios resembling real proteome data, we generated 45 different simulated scenarios with varying effect sizes, longitudinal trends, and variance levels based on proteome data by Liu et al. (2018). For each scenario, we then applied the approaches using a training dataset of 10, 50, or 1000 individuals with 10 time points covering a time range of 15 years. Each model was tested in an additional test dataset simulated under the same scenario, including varying lengths of follow‐up series from the baseline onwards (5, 10, or 15 years) to predict the risk of the event at the end of the maximum follow‐up time (20 years). The area under the receiver operating characteristic curve (AUROC) was used to measure the performance. Additionally, we evaluated each method using a simulated dataset with 1000 individuals and only 3 time points spanning 15 years, in order to assess the impact of reduced sampling points on model performance. In this dataset, all individuals had a follow‐up series of 15 years, ensuring at least 3 data points were available for model training.

**FIGURE 2:**
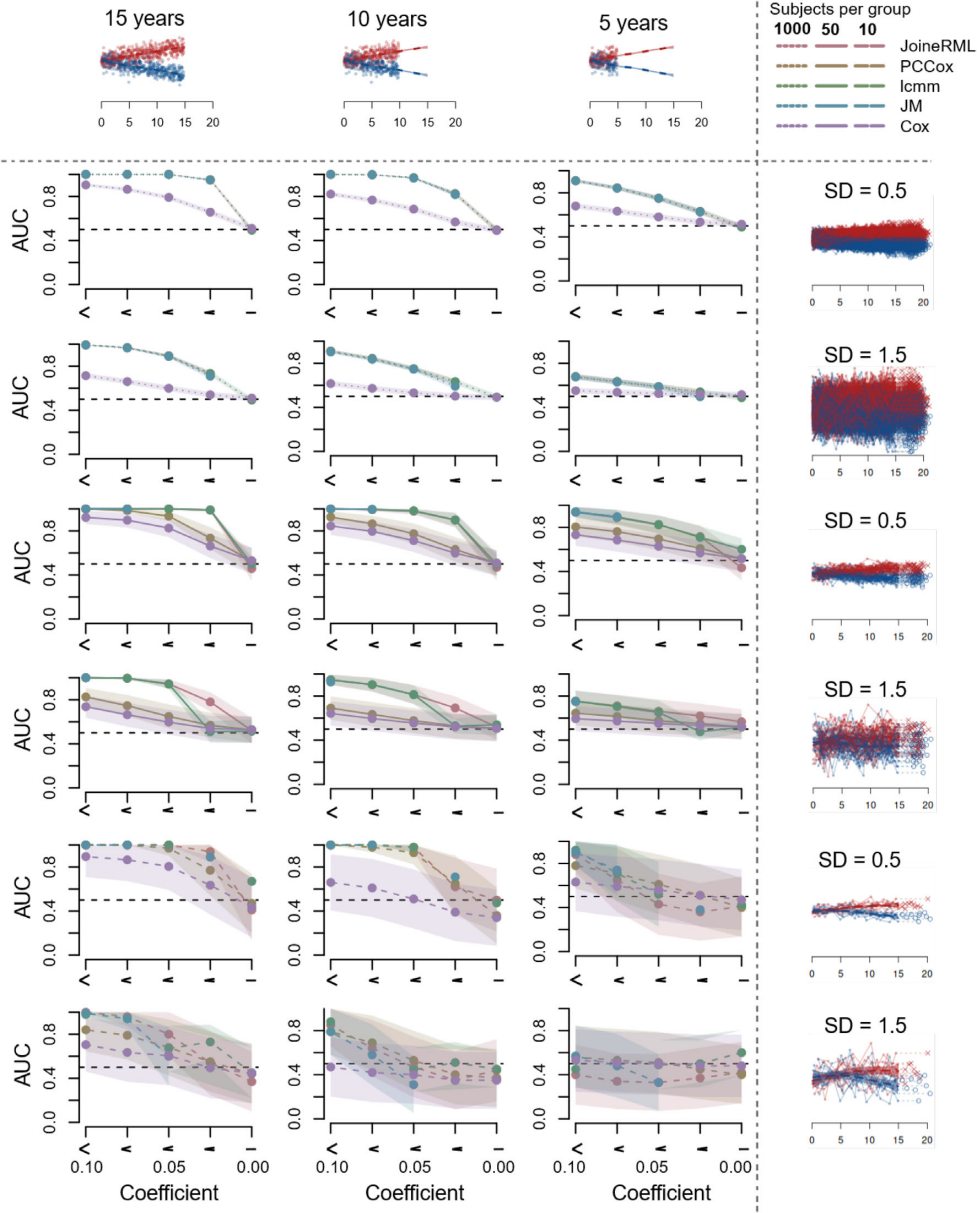
Performance of different joint modeling methods for jointly modeling longitudinal and time‐to‐event data in simulated test datasets. Four joint modeling methods (joineRML, JM, lcmm, PCCox) were tested along with the Cox proportional hazards model (CoxPH) as a baseline reference model. The areas under the receiver operating characteristic curves (AUROC) for each method are shown in each panel, with decreasing effect size along the *x*‐axis. The three columns correspond to varying lengths of the follow‐up series (5, 10, or 15 years) considered to predict the risk of the event at the end of the maximum follow‐up time (20 years). The rows correspond to different levels of variance in the data, with the top two having 1000 subjects per group (dotted lines), the middle two having 50 subjects per group (solid lines), and the bottom two having 10 subjects per group (dashed lines). The highlighted area represents the 95% confidence interval of the AUC values.

**FIGURE 3:**
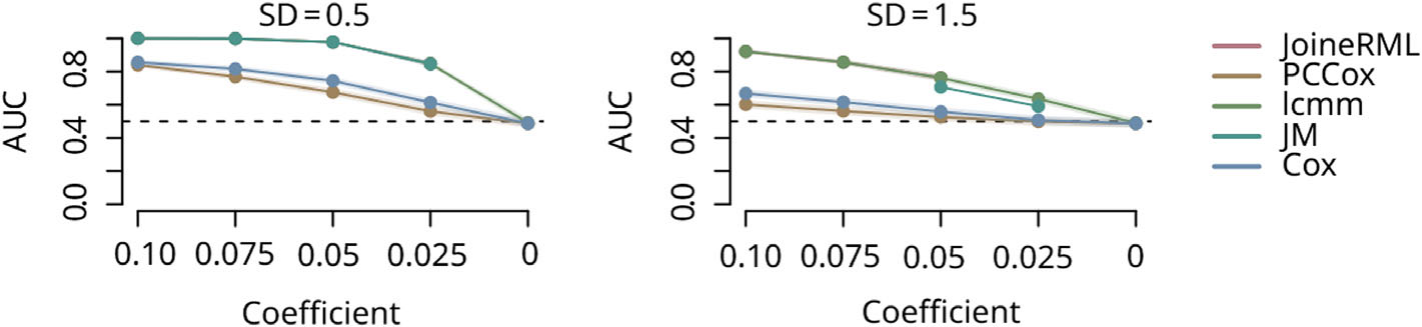
Performance of different joint modeling methods in simulated scenarios with short time‐series length. Four joint modeling methods (joineRML, JM, lcmm, PCCox) were tested along with the Cox proportional hazards model (CoxPH) as a baseline reference model in a simulation setting with only 3 data points spanning 15 years from 1000 simulated subjects. The areas under the receiver operating characteristic curves (AUROC) for each method are shown in each panel, with decreasing effect size along the *x*‐axis with 95% confidence interval of the AUC values in the highlighted area.

As expected, the performance of all the methods decreased when a shorter follow‐up was available for the prediction (Figure 2, columns). In these cases, predictions were sometimes close to or even worse than random chance, particularly when the number of individuals in the dataset was small, leading to AUROC values around or below 0.5. In line with this, the baseline Cox models using data from only a single time point showed systematically the lowest AUROC values (Wilcoxon signed‐rank test *p* <0.001). Similarly, the performance of the methods decreased with smaller effect sizes (Figure 2, *x*‐axes) or higher variance levels (Figure 2, rows), as expected, resulting in AUROC values around 0.5 when the signal in the data was non‐existent (i.e., when the effect size coefficient was 0). The impact of the decreasing effect size was more pronounced when the variance levels were higher. The performance also decreased when the number of individuals available for model training decreased (solid versus dashed lines, *p* <0.001). This was particularly true when only a short follow‐up was available (Figure 2, rightmost column). In the simulation study with limited longitudinal follow‐up measurements (Figure 3), both COX and PCCox consistently showed the lowest performance, while lcmm, JoineRML, and JM performed similarly when their models converged.

In general, joineRML and lcmm showed the best performance, with joineRML tolerating the increased variance levels better than lcmm. JM performed similarly to joineRML and lcmm when the model fitting converged, but unlike the other methods, it left many scenarios (~30%) without any result mostly due to reaching the iteration limit without convergence in data sets with high variance (see Supplementary Table S1 for more detailed breakdown of the scenarios and error messages). This was true, especially with the more challenging settings of small effect sizes or high variance levels. The PCCox models PCCox had generally the worst performance among the methods jointly modeling longitudinal and time‐to‐event data. However, PCCox suffered least when the number of individuals in the model training decreased, making it a plausible additional option in such settings. Particularly with the more challenging simulation scenarios, all the JM approaches performed significantly better than the baseline reference model CoxPH (*p* <0.001).

We additionally tested JMbayes2 (version 0.5–7) (Rizopoulos et al., 2026) on the same simulated scenarios. With JMbayes2, we noticed unexpected performance drops in cases where the other methods performed well (Supplementary Figure S2 Top row, highest coefficients) that we were not able to find the cause for, but on the other hand, JMbayes2 did perform relatively well on most of the simulation settings. In the simulation study with limited longitudinal follow‐up measurements, JMbayes2 displayed a counterintuitive downward trend in predictive performance as the signal in the data increased (Supplementary Figure S3). This pattern may suggest a potential error in the usage of JMbayes2, though our attempts to identify the specific cause were unsuccessful. For this reason, we decided to only include the results of JMbayes2 in the Supplementary of this manuscript.

In addition to differences in the prediction accuracy, there were also some differences in the training times required by the different modeling approaches. JMbayes2 required the longest time to train averaging 5900 s in the simulated data sets containing 1000 subjects, JoineRML was the second most computationally heavy method at 1576 s, lcmm required the time of 87 s, followed by JM at 11 s, while PCCox only took a fraction of a second to train. Overall, our results suggest that the performance of the different modeling approaches varies depending on the simulated scenario, highlighting the need for selecting an appropriate approach depending on the specific characteristics of the data being studied. In our study, we specifically evaluated the impact of effect size, length of follow‐up series, and variance to provide insights into which methods generally perform well across most scenarios (Figure 2). To further refine model selection, researchers could simulate ground‐truth data that closely resembles their data or utilize domain knowledge, such as previously validated proteins, to guide the selection.

### 2.2 JM reveals proteins associated with type 1 diabetes progression

To study the applicability of JM in a real‐world setting, we applied the top‐performing approaches from the simulations, joineRML and lcmm, to proteomics data on early human type 1 diabetes development from the study by Liu et al. (2018). Additionally, we tested the utility of the PCCox modeling PCCox. The data consisted of blood plasma protein profiles across 2084 proteins from 21 children, including 11 children who developed type 1 diabetes and 10 matched control children. The follow‐up time points spanned from 0.8 to 14.4 years of age or until the onset of the disease, covering a total of nine time points. Among the children progressing to the disease, the average age of disease onset was 11.7 years (range 8.5–13.7 years). The original study used regression models to assess temporal trends in protein abundances over time. In contrast, the JM approaches applied here provide a fundamentally different framework, allowing us to assess associations between longitudinal protein abundances and time to diagnosis. Specifically, we aimed to identify proteins that could predict the probability of disease onset at 13 years of age, using follow‐up measurements by 5, 6, 7, or 8 years of age. The potential of the models to stratify individuals into high‐ and low‐risk groups was evaluated using AUROC values in the same data.

**FIGURE 4:**
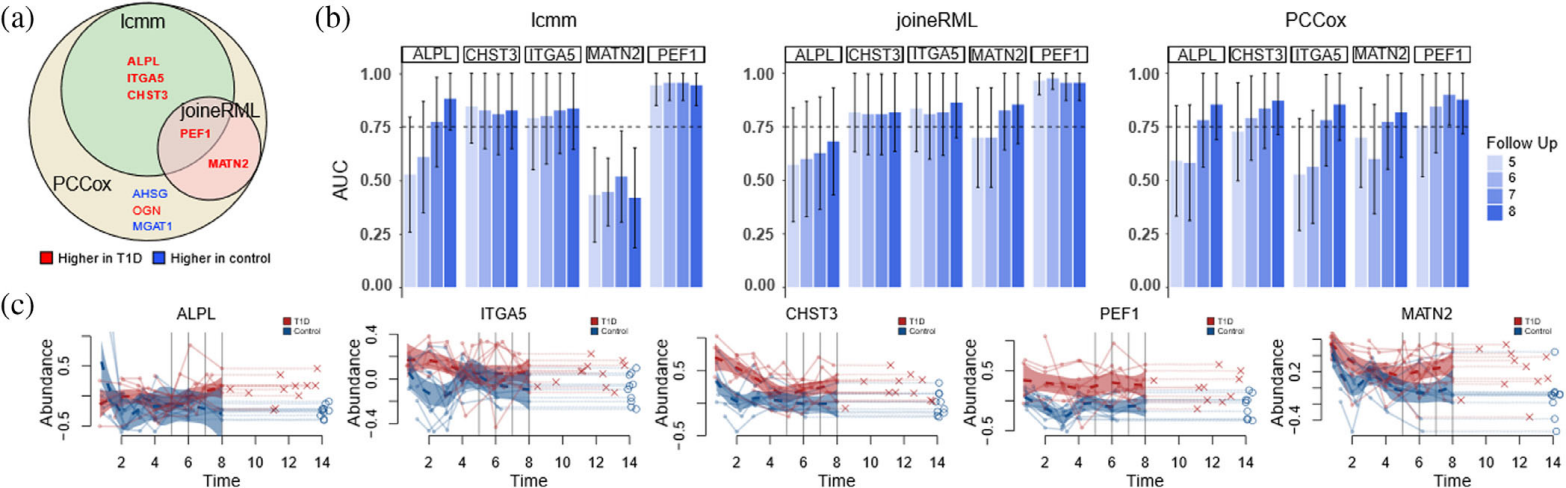
Joint modeling of longitudinal and time‐to‐event data for dynamic prediction of type 1 diabetes onset using proteomics. (a) Top candidate proteins identified using the joint modeling approaches of joineRML and lcmm or the partly conditional Cox modeling PCCox. Proteins with increased levels associated with type 1 progression are highlighted in red, and proteins with decreased levels are highlighted in blue. (b) Areas under the receiver operating characteristic curves (AUROC) of the seven proteins that were identified by at least two of the modeling approaches, using the follow‐up measurements by 5, 6, 7, or 8 years of age to predict the risk of the disease onset at 13 years of age with 95% confidence intervals. (c) Protein abundance profiles of proteins associated with the risk of type 1 diabetes onset.

To align with the filtering criteria of the original study, we focused on proteins identified by at least six unique peptides and at most 10 missing values over all samples. Using this approach, JoineRML identified 10 proteins with nominally significant association with the disease risk (Wald test *p*< 0.05), of which two had measurements from at least five individuals in both classes and AUROC >0.75 in at least the two last evaluation points (Figure 4a). Lcmm identified 26 proteins with two latent classes by the Bayesian information criterion associated with the disease onset, constraining that both classes had more than five individuals. Of these proteins, four had AUROC >0.75 in at least the two last evaluation points. PCCox identified 20 proteins associated with the disease onset (*p*< 0.01), of which eight had measurements from at least five individuals in both classes and AUROC >0.75 in at least the two last evaluation points. Although PCCox identified the largest number of candidate proteins, it should be noted that its performance in the simulation study suggested a higher likelihood of false positives, illustrating that a larger candidate list does not necessarily imply better predictive performance.

In total, five proteins (ALPL, ITGA5, CHST3, PEF1, MATN2) were found to be associated with the risk of disease onset by at least two different JM approaches, all of which showed increased abundance in children progressing to type 1 diabetes (Figure 4a–c). These proteins could be further divided into those that separated the groups independent of time (CHST3, PEF1) and those that had a time‐varying trend (ALPL, ITGA5, MATN2), as seen by increased stratification accuracy only at later time points during the follow‐up (Figure 4b). None of these proteins were reported in the original study (Liu et al., 2018) to show different temporal trends between the children developing type 1 diabetes and the healthy controls. This is expected, as the JM approach focuses on capturing associations between longitudinal protein changes and time‐to‐diagnosis rather than solely analyzing temporal trends.

While these results are encouraging, further validation in independent cohorts is necessary. Nevertheless, our findings demonstrate the potential of JM to identify new candidate proteins that could predict the future development of type 1 diabetes even years before the clinical diagnosis. As the methods were trained using the whole data set, we assessed the risk of overfitting by performing leave‐one‐out cross‐validation (LOO‐CV) on the selected proteins. For each iteration, models were trained on all samples except one, with predictions made for the left‐out sample. After repeating this process for every sample, the resulting predictions were used to calculate the AUC values. The results of this LOO‐CV can be found in the supplementary (Supplementary Figure S1). The figure shows that lcmm seems to be the most prone to overfitting, which may stem from its use of grid search to optimize its parameters. The other two methods (joineRML, PCCox) did not see notable performance drop‐offs in the cross‐validation study.

## 3 DISCUSSION

Over the past few decades, there have been several advances in methods for analyzing and predicting time‐to‐event outcomes from longitudinal clinical data. However, the application of these methods to proteomics and other omics data has not been explored extensively. To address this gap, we evaluated various approaches for JM of longitudinal and time‐to‐event data, both in simulated and real settings, and presented a JM strategy for longitudinal proteomics studies (Figure 1).

Due to the variety of different types of joint longitudinal models, previous studies have mostly focused on comparing differences in their parameter estimates (Rappl et al., 2021; Yuen & Mackinnon, 2016). However, in this study, we were particularly interested in assessing the predictive accuracy of the models and their utility for high‐dimensional proteome datasets. Furthermore, we wanted to assess the robustness of model fitting and options for automated model selection. While all the methods were technically feasible, considerable differences were observed among them in terms of their accuracy, convergence, and significance estimates.

Our results in various simulated datasets showed that joint models were more accurate than conventional CoxPH models in predicting individual outcomes. The benefits were largest with more challenging scenarios, involving only subtle differences over time or increased variation. However, the performance of joint models varied depending on the approach used, with significant differences in the prediction accuracies between the approaches. These results are in line with previous studies showing that the choice of modeling approach can have a significant impact on performance, especially when the complexity of the data increases (Rappl et al., 2021; Suresh et al., 2017). In general, our results suggested that joineRML and lcmm performed most consistently across all the simulated datasets.

As indicated in previous studies, the use of JM calls for careful consideration of the model types, parameters, and association structures that best capture the relationship between the longitudinal process and the event time (Hickey et al., 2016, 2018a; Papageorgiou et al., 2019). The most appropriate modeling approach may vary depending on the research question and the data being used. Many individual models could likely be improved by optimizing the parameters and association structures best suited for the data at hand. However, when dealing with proteomics datasets that include hundreds or thousands of proteins, manual model selection is often impractical, making model selection automation necessary. The methods compared in this study provided different types of modifications for the parameters and model specification, with some having nearly unlimited flexibility to fit complex models to the data, while others had more restrictions. The lcmm framework also included a grid search option for estimating the model to avoid convergence toward a local optimum.

Longitudinal models are by design flexible in capturing trends over time, and as a result, their predictions are refined by incorporating more time points in the data. This was clear when the JM approach was applied on the real proteomics dataset on early development of type 1 diabetes. Consistent with the simulation results, proteins that were well separated already at the beginning of the longitudinal measurements were able to differentiate between individuals who later developed type 1 diabetes and healthy controls already at the early time points. However, proteins that only separated the groups over time were not detected by the model until at later time points.

Type 1 diabetes is an autoimmune disease where various genetic and environmental factors are linked to the destruction of insulin‐producing pancreatic *β*‐cells. Prior studies have identified serum and plasma proteins as potential early indicators of the disease by examining differences in protein levels at single or multiple time points (Liu et al., 2018; Moulder et al., 2015). For example, the study by Liu et al. (2018) used mixed effects modeling, focusing on proteins that had significant interaction effects between time and the study group (case/control). The JM of both longitudinal and time‐to‐event data applied here allows for the association of protein abundance changes with the prediction of event time, which is particularly important for diseases like type 1 diabetes that typically involve a long symptom‐free period before clinical signs appear. Although the first appearance of type 1 diabetes‐associated autoantibodies is currently considered the first sign of the disease, the time between their appearance and clinical manifestation can vary greatly. Therefore, discovery of early indicators of the disease is crucial for the development of future therapeutic and preventive strategies. Our results suggest that JM could reveal new candidate proteins beyond using the longitudinal data alone, which could enable predicting the development of the disease even before the appearance of autoantibodies, years before clinical diagnosis.

While different approaches are expected to produce different results depending on the specific goals of the analysis, findings detected by multiple tools addressing the same question are often the most interesting and reliable. Interestingly, all the five proteins identified by at least two different methods were supported by previous research to be associated with type 1 diabetes. Three of the proteins were related to the extracellular matrix, cell adhesion, and migration. Matrilin 2 (MATN2) belongs to the matrilin family of proteins that are associated with the formation of filamentous networks within the extracellular matrix, including the interstitial matrix in the insulin‐producing pancreatic islets of the *β*‐cells (Morgan & Richardson, 2018). Similarly, integrin alpha‐5 (ITGA5), a member of the integrin family, is associated with the cell membrane fibronectin receptor, facilitating cell‐to‐cell and cell‐to‐extracellular matrix adhesion (Hynes, 2002). Carbohydrate sulfotransferase 3 (CHST3) is related to the enzyme that catalyzes the sulfation of proteoglycans, namely chondroitin, located in the extracellular matrix and associated with cell migration (Haylock‐Jacobs et al., 2011). Recently, dysregulation related to the extracellular matrix, cell adhesion, and cell migration within the pancreas has been associated with type 1 diabetes (Buchacher et al., 2022; Välikangas et al., 2022). In general, integrin‐mediated signaling has been suggested to be crucial for *β*‐cell survival (Arous & Wehrle‐Haller, 2017). Moreover, a high‐glucose environment has been shown to induce the expression of MATN2 in mouse mesangial cells and potentially play a role in diabetic nephropathy (Zhang et al., 2016), while the genetic locus of CHST3 has been shown to significantly influence insulin secretion in nondiabetic Caucasian individuals (‘t Hart et al., 2013). Increased blood serum levels of alkaline phosphatase, tissue‐nonspecific (ALPL), an enzyme essential to mineralization, have been associated with type 1 diabetes both recently and already many decades ago (Herzog et al., 2021; Maxwell et al., 1986). It has also been linked to new‐onset diabetes in hypertensive adults (Zhang et al., 2020). Related, peflin (PEF1) is a calcium binding protein (Kitaura et al., 1999), and interestingly, calcium metabolism and increased calcium levels in many tissues have been linked to diabetes (Levy et al., 1994).

However, the relatively small number of individuals is a major limitation of this study and, therefore, careful further validation in larger cohorts would be needed to confirm the utility of the identified proteins. Another limitation of this study is the use of an already relatively old dataset, which, while offering valuable longitudinal proteomic information, may not have the same quality and resolution as more recent datasets generated with advanced mass spectrometry instruments. Additionally, although we tested various modeling approaches under different simulated conditions, the results may be affected by the specific characteristics of the dataset, such as the small sample size and noise in the data. Although all the proteins identified using joint models were well supported by existing literature, no additional validations were performed in this study. Further validation with newer datasets and larger cohorts would be necessary to confirm the robustness and generalizability of our findings. We acknowledge that the results from the real‐world case study may be overly optimistic, as all methods were trained using the entire dataset due to the small sample size, which increases the risk of overfitting. This limitation could be mitigated in future analyses by using a larger dataset that would allow for a more robust split into training and testing sets.

In conclusion, we presented here a JM strategy for longitudinal proteomics studies and demonstrated its utility for predicting the dynamic disease risk of an individual from longitudinal proteome profiles, using both simulated and real data. Through benchmarking of various JM methods in simulated datasets with varying complexities, we demonstrated the advantages of longitudinal methods over single time point studies. Our approach was further supported by a re‐analysis of a real proteomics study of early type 1 diabetes prediction, where multiple early candidate proteins associated with disease onset were identified. The discovery of such early disease indicators could lead to new opportunities for future therapeutic developments by allowing for improved clinical trials and by increasing the window for intervention. To fully realize the potential of the JM methods, the field would greatly benefit from longitudinal datasets with more subjects and measurements obtained using newer high‐resolution instruments.

## 4 METHODS

### 4.1 The JM framework

We investigated four different approaches for predicting time‐to‐event outcomes from longitudinal data. These included maximum likelihood‐based JM (Rizopoulos, 2010), JM based on latent Gaussian processes (joineRML) (Hickey et al., 2018b), joint lcmm (Proust‐Lima et al., 2016), and PCCox models (PCCox) (Maziarz et al., 2017; Zheng & Heagerty, 2005). Each method differs in how they capture the association between longitudinal and time‐to‐event outcomes. The maximum likelihood‐based JM framework (Rizopoulos, 2010) typically employs shared random effects to link the repeated measurements with the survival process, allowing for the evaluation of both current value and slope associations. The latent Gaussian process model (joineRML (Hickey et al., 2018b)) accommodates flexible, possibly nonlinear associations through latent process structures. Joint lcmm (Proust‐Lima et al., 2016) facilitate the identification of distinct patient subgroups via latent classes. The PCCox model offers an approach to model the longitudinal‐survival association by predicting risk for time intervals. For further details we refer the reader to the original publications and introduce here a common notation for joint models instead.

A general form of a linear mixed effects model can be described as follows:

(1) $$y\left(t\right)=X_{L}\left(t\right)\beta_{L}+Z\left(t\right)b+\varepsilon \left(t\right)$$

Here, *y*(*t*) denotes the observed longitudinal measurements at time *t*, *X*_*L*_(*t*) and *Z*(*t*) represent the covariates for the fixed effects β_*L*_ and the random effects *b*, respectively, and the error terms ε(*t*) are assumed to follow a normal distribution *N*(0, σ ^2^). A common choice for the time‐to‐event part is the CoxPH model:

(2) $$h\left(t\right)=h_{0}\left(t\right)\exp\left\{X_{E}\left(t\right)\beta_{E}+W\left(t\right)\right\}$$

where *h*_0_(*t*) is the baseline hazard, *X*_*E*_(*t*) represents the covariates for the fixed effects β_*E*_, and *W*(*t*) is the association structure to couple the linear mixed effects model with the Cox propositional hazards function. The form of the association structures varies between different types of models and is thoroughly reviewed, for instance, by Hickey et al. (2016).

In this study, we used four R packages that implement these different modeling approaches: *JM* version 1.5–2 (Rizopoulos, 2010), *joineRML* version 0.4.5 (Hickey et al., 2018b), lcmm version 2.0.0 (Proust‐Lima et al., 2017), and partlyconditional version 2.0 (Maziarz et al., 2017; Zheng & Heagerty, 2005). As a baseline reference model, we used the standard CoxPH model with data from the last available value from the longitudinal profiles, implemented in the R package survival version 3.3–1 (Therneau & Grambsch, 2000).

### 4.2 Type 1 diabetes proteomics data

We reanalyzed the proteome profiling data from the study by Liu et al. (2018). The data consisted of plasma proteome profiles obtained from 11 children progressing to type 1 diabetes and 10 matched control children. The profiles included nine longitudinal measurements, which covered the period from birth to the onset of type 1 diabetes for the individuals who developed the disease, and matched profiles from healthy control children. The samples were analyzed with a TMT‐10plex‐based LC–MS/MS approach using a Q Exactive HF mass spectrometer. All nine longitudinal measurements from one individual were analyzed in a single run together with a reference sample pooled from all the samples in the experiment. The data were preprocessed similarly as in the original study, which involved standardizing all reporter ion intensities using the pooled reference sample, log_2_‐transforming the data, and normalizing it with median normalization. We only included non‐depleted proteins in our analysis, which resulted in a total of 2084 proteins for the analysis. The selected methods were trained for each protein separately.

### 4.3 Simulated datasets

As a starting point for our simulations, we used the average expression levels and standard deviations from the Liu et al. proteomics dataset. A total of 100 subjects (50 cases and 50 controls) were simulated to have 10 follow‐up time points evenly distributed over a time‐span of 15 years. Events were simulated to occur after the follow‐up period, with a mean of 18 years and a standard deviation of 1 years. To simulate expression levels under various scenarios, we obtained overall expression and its variation from normal distribution with a mean (on a log‐scale) expression of 6 and a standard deviation of 0.5 to 1.5, in intervals of 0.5. To simulate longitudinal changes in the differences between cases and controls, we changed the slope coefficients, which were positive for cases and negative for controls, from 0 to ±0.1, in intervals of 0.025. Finally, to account for random intercepts, individual differences were simulated by adding values drawn from a normal distribution with a standard deviation of 0.1. For each scenario, we simulated one dataset for training and another for testing the models.

### 4.4 Model fitting

A linear mixed effects model was fitted for each protein to capture the individual longitudinal trajectories over the follow‐up time, allowing for random intercepts and random slopes per subject through time, using the formula: *proteinAbundance ~ time + (time|individual)*. These were combined with a CoxPH model using the different joint modeling R packages to estimate the association of the protein with the risk of disease onset. No time‐independent covariates were considered, resulting in the baseline time‐to‐event models of the form *Surv(eventTime, outcome) ~* 1, where the outcome refers to the disease status at the end of the follow‐up study, and *eventTime* is the time of the disease diagnosis or the last follow‐up observation. The significance of the association between the linear mixed effect model and the Cox model was determined using the Wald test. The *p*‐values were adjusted for multiple testing using the Benjamini–Hochberg method to control the false discovery rate.

The lcmm models were estimated using a Weibull hazard as the baseline risk function, assuming proportional hazards across the latent classes. As recommended by the authors of lcmm (Proust‐Lima et al., 2016, 2017), the starting parameters for lcmm were searched for by a grid search starting from a model fitted with one (ng = 1) to two latent classes (ng = 2).

The conventional CoxPH model was defined as *coxph(Surv(eventTime,outcome) ~ proteinAbundance*, where the *proteinAbundance* was the abundance of the protein at the last time point of the follow‐up series.

### 4.5 Evaluation of the model performance in the simulated datasets

The overall performance of the different joint models was evaluated by assessing how well the models could predict the correct status of an individual at the end of the study (20 years). This was done by considering different lengths of follow‐up times (5, 10, or 15 years). In each case, an independent dataset was used for the evaluation, which was generated from the same simulation model as the original training data for the model. The performance was measured as the AUROC, estimated using the R package pROC (version 1.18.0).

### 4.6 Identification of proteins associated with the risk of type 1 diabetes

The predictive ability of each protein in the proteomics dataset by Liu et al. (Liu et al., 2018) was evaluated using varying lengths of longitudinal follow‐up times from 5 to 8 years of age, with an interval of 1 year. Similar to the simulated datasets, the ability of each protein to predict the disease status of an individual at the end of the study (13 years) was quantified using AUROC. This allowed us to identify proteins that were predictive of the disease risk already at the early time points as well as those that predicted the outcome only after more information had been collected. To focus on the most promising findings, only proteins with an AUROC of 0.75 or higher in at least the last two evaluation points (7 and 8 years) were included in the results. Furthermore, we only considered proteins identified by at least six unique peptides and at most having 10 missing values over all samples.

## AUTHOR CONTRIBUTIONS

**Tommi Välikangas:** Data curation; formal analysis; writing – review and editing. **Markus Lindén:** Software; data curation; writing – review and editing; investigation; visualization. **Laura L. Elo:** Conceptualization; formal analysis; methodology; supervision; writing – review and editing. **Tea Ammunét:** Software; writing – original draft; writing – review and editing; investigation; data curation. **Tomi Suomi:** Data curation; software; formal analysis; supervision; visualization; methodology; writing – review and editing.

## FUNDING INFORMATION

LE reports grants from the European Research Council ERC (677943), European Union's Horizon 2020 research and innovation programme (955321), Academy of Finland (310561, 314443, 329278, 335434, 335611, and 341342), and Sigrid Juselius Foundation during the conduct of the study. ML has been supported by the Vilho, Yrjö and Kalle Väisälä Foundation. Our research is also supported by Biocenter Finland, and ELIXIR Finland.

## CONFLICT OF INTEREST STATEMENT

The authors declare that there is no conflict of interest.

## Supporting information


**Supplementary Figure S1.** Performance of different joint modeling methods for jointly modeling longitudinal and time‐to‐event data in simulated test datasets. Five joint modeling methods (joineRML, JM, lcmm, PCCox, JMbayes2) and the Cox proportional hazards model were tested. The AUROC‐values for each method are shown in each panel, with decreasing effect size along the *x*‐axis. The columns correspond to varying lengths of the follow‐up series considered to predict the risk of the event at the end of the maximum follow‐up time (20 years). The rows correspond to different levels of variance in the data, with the top two having 1000 subjects per group (dotted lines), the middle two having 50 subjects per group (solid lines) and the bottom two having 10 subjects per group (dashed lines). The highlighted area represents 95% confidence interval of the AUC values.
**Supplementary Figure S2.** Performance of different joint modeling methods in simulated scenarios with short time‐series length. Five joint modeling methods (joineRML, JM, lcmm, PCCox, JMbayes2) were tested along with the Cox proportional hazards model (CoxPH) as a baseline reference model in a simulation setting with only 3 data point spanning 15 years from 1000 simulated subjects. The areas under the receiver operating characteristic curves (AUROC) for each method are shown in each panel, with decreasing effect size along the *x*‐axis with 95% confidence interval of the AUC values in the highlighted area.
**Supplementary Figure S3.** Areas under the receiver operating characteristic curves (AUROC) of the five proteins with follow‐up measurements by 5, 6, 7, or 8 years of age to predict the risk of the disease onset at 13 years of age using LOO‐CV and (A) lcmm, (B) joineRML and (C) PCCox.

**Supplementary Table S1.** Detailed breakdown of the scenarios and error messages.

## Data Availability

No datasets were generated or analysed during the current study. We used published data (supplementary of: https://doi.org/10.1016/j.jprot.2017.10.004). As for the simulated data, the simulation protocols are available through the GitHub link provided in the manuscript.
