# Supplementary material for: Joint modeling of longitudinal and time‐to‐event data for dynamic disease risk prediction using proteomics
Source: Protein Sci. 2026 May 18;35(6):e70621. doi: 10.1002/pro.70621 (PMC13182269; doi:10.1002/pro.70621)
Supplement: Supplementary file 1 — Supplementary Figure S1. Performance of different joint modeling methods for jointly modeling longitudinal and time‐to‐event data in simulated test datasets. Five joint modeling methods (joineRML, JM, lcmm, PCCox, JMbayes2) and the Cox proportional hazards model were tested. The AUROC‐values for each method are shown in each panel, with decreasing effect size along the x‐axis. The columns correspond to varying lengths of the follow‐up series considered to predict the risk of the event at the end of the maximum follow‐up time (20 years). The rows correspond to different levels of variance in the data, with the top two having 1000 subjects per group (dotted lines), the middle two having 50 subjects per group (solid lines) and the bottom two having 10 subjects per group (dashed lines). The highlighted area represents 95% confidence interval of the AUC values. Supplementary Figure S2. Performance of different joint modeling methods in simulated scenarios with short time‐series length. Five joint modeling methods (joineRML, JM, lcmm, PCCox, JMbayes2) were tested along with the Cox proportional hazards model (CoxPH) as a baseline reference model in a simulation setting with only 3 data point spanning 15 years from 1000 simulated subjects. The areas under the receiver operating characteristic curves (AUROC) for each method are shown in each panel, with decreasing effect size along the x‐axis with 95% confidence interval of the AUC values in the highlighted area. Supplementary Figure S3. Areas under the receiver operating characteristic curves (AUROC) of the five proteins with follow‐up measurements by 5, 6, 7, or 8 years of age to predict the risk of the disease onset at 13 years of age using LOO‐CV and (A) lcmm, (B) joineRML and (C) PCCox. [file PRO-35-e70621-s002.docx]

Joint modeling of longitudinal and time-to-event data for dynamic disease risk prediction using proteomics

Markus Lindén 1, Tea Ammunét 1, Tommi Välikangas 1, Laura L. Elo 1,2,*, Tomi Suomi 1,*,#

1 Turku Bioscience Centre, University of Turku and Åbo Akademi University, FI-20520 Turku, Finland

2 Institute of Biomedicine, University of Turku, FI-20520 Turku, Finland

* Shared last author

# Correspondence: [tomi.suomi@utu.fi](mailto:tomi.suomi@utu.fi)

**Suplementary data**


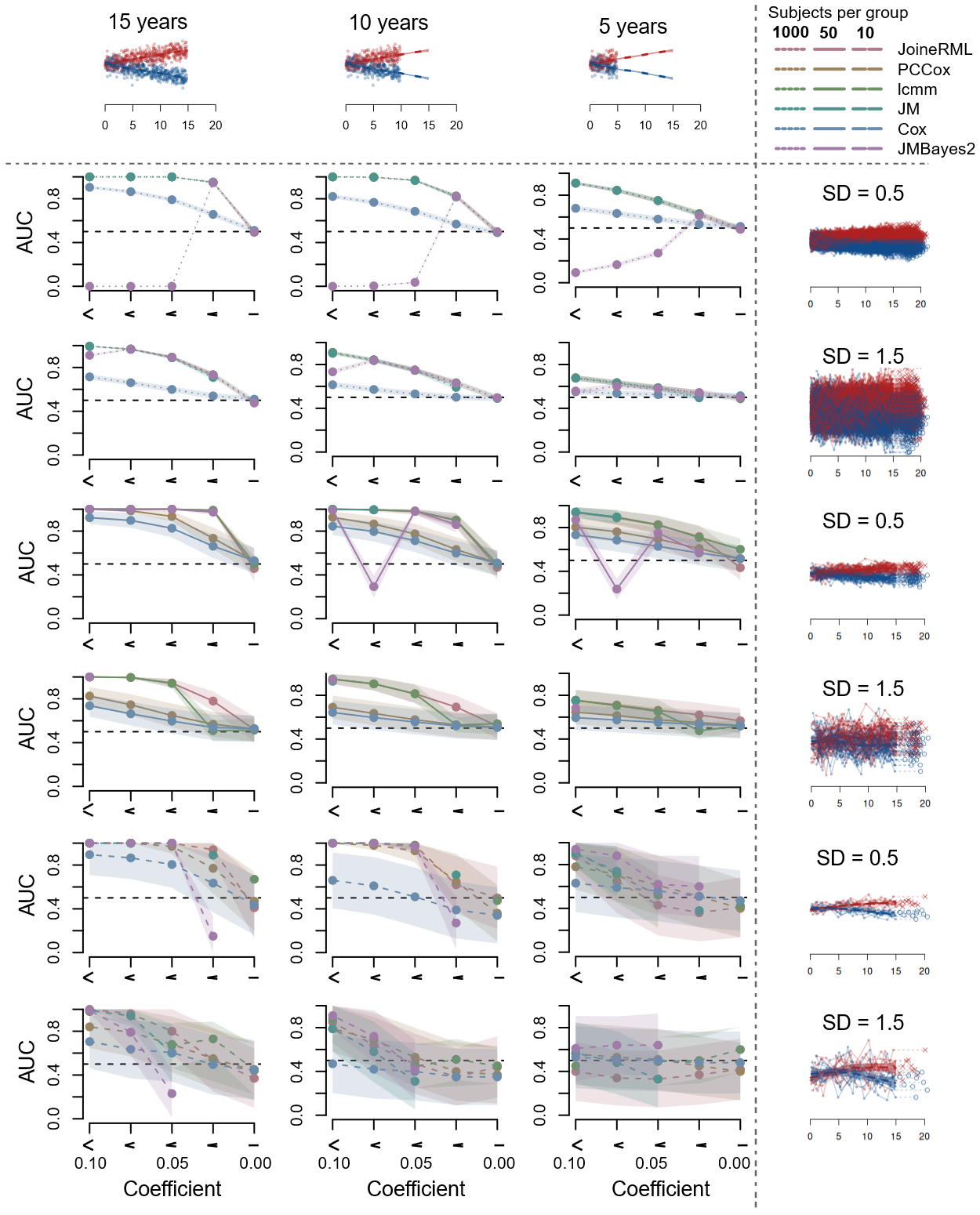


**Supplementary Figure S1.** **Performance of different joint modeling methods for jointly modeling longitudinal and time-to-event data in simulated test datasets.** Five joint modeling methods (joineRML, JM, lcmm, PCCox, JMbayes2) and the Cox proportional hazards model were tested. The AUROC-values for each method are shown in each panel, with decreasing effect size along the x-axis. The columns correspond to varying lengths of the follow-up series considered to predict the risk of the event at the end of the maximum follow-up time (20 years). The rows correspond to different levels of variance in the data, with the top two having 1000 subjects per group (dotted lines), the middle two having 50 subjects per group (solid lines) and the bottom two having 10 subjects per group (dashed lines). The highlighted area represents 95% confidence interval of the AUC values.

**
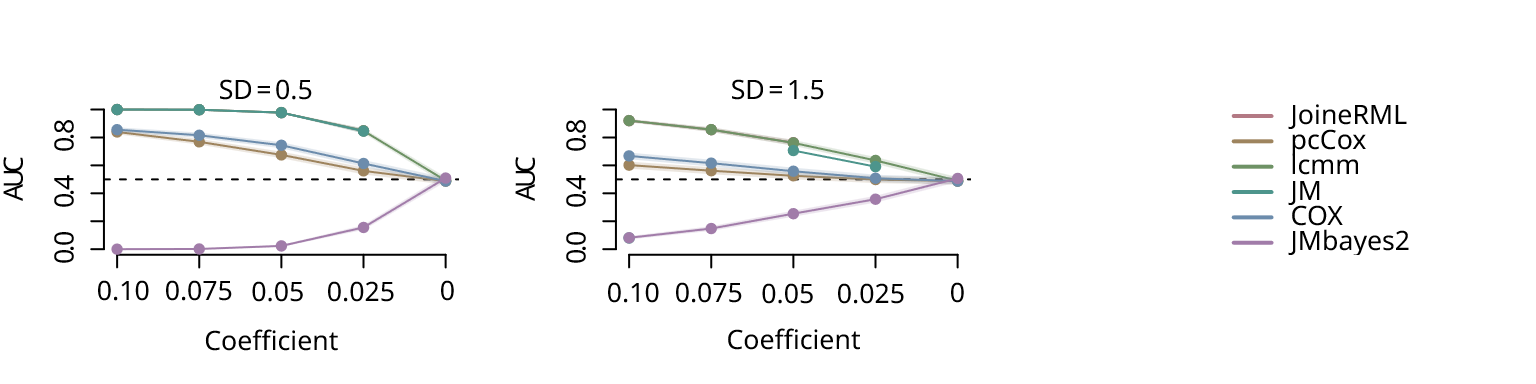
Supplementary Figure S** **2**. **Performance of different joint modelling methods in simulated scenarios with short time-series length.** Five joint modeling methods (joineRML, JM, lcmm, PCCox, JMbayes2) were tested along with the Cox proportional hazards model (CoxPH) as a baseline reference model in a simulation setting with only 3 data point spanning 15 years from 1000 simulated subjects. The areas under the receiver operating characteristic curves (AUROC) for each method are shown in each panel, with decreasing effect size along the x-axis with 95 % confidence interval of the AUC values in the highlighted area.


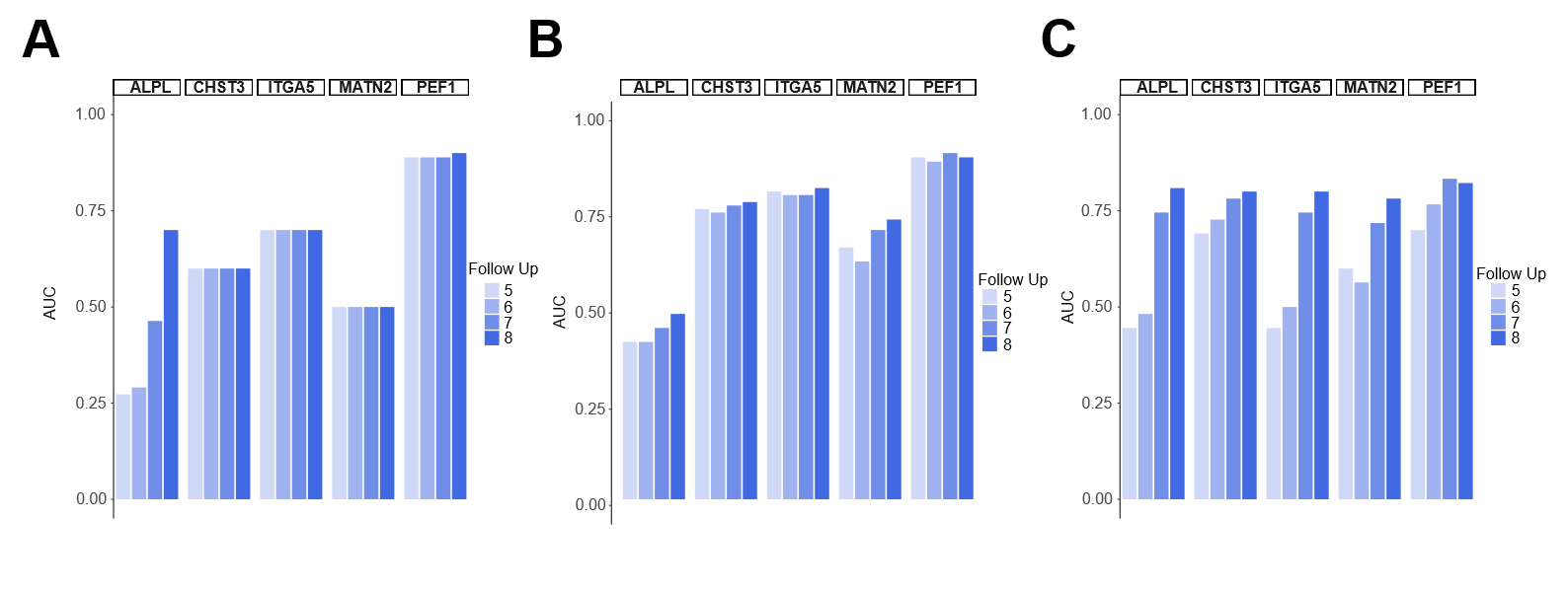


**Supplementary Figure S3**. Areas under the receiver operating characteristic curves (AUROC) of the five proteins with follow-up measurements by 5, 6, 7, or 8 years of age to predict the risk of the disease onset at 13 years of age using LOO-CV and **A)** lcmm, **B)** joineRML and **C)** PCCox.
